# Immunoglobulin-like transcript 2 (ILT2) is a biomarker of therapeutic response to oncolytic immunotherapy with vaccinia viruses

**DOI:** 10.1186/2051-1426-2-1

**Published:** 2014-01-27

**Authors:** Andrew Zloza, Dae Won Kim, Seunghee Kim-Schulze, Michael C Jagoda, Vladia Monsurro, Francesco M Marincola, Howard L Kaufman

**Affiliations:** 1 Rush University Cancer Center, 1725 West Harrison Street, Chicago, IL 60612, USA; 2 Department of Immunology/Microbiology, Rush University Medical Center, 1750 West Harrison Street, Chicago, IL 60612, USA; 3 The Tumor Immunology Laboratory, Department of Surgery, Mount Sinai School of Medicine, One Gustave L. Levy Place, New York, NY 10029, USA; 4 Infectious Disease and Immunogenetics Section, Department of Transfusion Medicine, Clinical Center, National Institutes of Health, 9000 Rockville Pike, Bethesda, MD 20892, USA; 5 Department of Pathology and Diagnostic, University of Verona Medical School, Verona, Italy; 6 Research Branch, Sidra Medical and Research Centre, Doha, Qatar; 7 Rutgers Cancer Institute of New Jersey, 195 Little Albany Street, New Brunswick, NJ 08903, USA

**Keywords:** Immunoglobulin-like transcript 2, Biomarker, Vaccinia, Oncolytic virus, Immunotherapy, T cells, Cancer vaccines

## Abstract

**Background:**

Oncolytic viruses represent a novel form of cancer immunotherapy. Vaccinia viruses encoding human T cell co-stimulatory molecules have demonstrated clinical activity in phase I clinical trials in patients with advanced melanoma. However, predictive biomarkers of therapeutic response have not yet been identified.

**Methods:**

A customized microarray was performed to identify changes in peripheral blood mononuclear cell (PBMC) gene expression upon exposure to recombinant oncolytic vaccinia viruses. Up-regulated and down-regulated genes were identified and selected for further analysis using PBMC samples from normal donors and oncolytic virus-treated patients before and after viral injection. Quantitative PCR and flow cytometry of defined T cell subsets was performed to evaluate expression patterns and clinical correlations.

**Results:**

The microarray identified 301 genes that were up-regulated and 960 genes that were down-regulated in T cells after exposure to oncolytic vaccinia virus. The B7.1 gene was highly up-regulated and the immunoglobulin-like transcript 2 (ILT2) gene was highly down-regulated by vaccinia-B7.1, which was consistent with the known inverse regulation of these two genes. We observed an inverse association between ILT2 expression in the tumor microenvironment and clinical response and further identified ILT2 as a marker of regulatory CD4+ and suppressor CD8+ T cell responses and whose down-regulation was predictive of therapeutic responses in patients treated with oncolytic virus immunotherapy.

**Conclusions:**

ILT2 is a new putative biomarker of T cell and clinical response in patients treated with oncolytic vaccinia virus immunotherapy. Further confirmation of ILT2 as a biomarker requires prospective validation in a larger series of clinical trials.

## Background

Oncolytic viruses mediate tumor regression through selective replication in and lysis of tumor cells and induction of systemic anti-tumor immunity [1]. Vaccinia viruses are members of the poxviridae family of DNA viruses and have been used to effectively prevent smallpox in humans, as a recombinant vector for gene delivery and systemic vaccination, and as an oncolytic immunotherapy [2]. We have previously demonstrated that administering recombinant vaccinia viruses encoding T cell co-stimulatory molecules by intra-tumoral injection induces objective clinical responses and improved local and systemic anti-tumor immunity in patients with metastatic melanoma [3,4]. In a phase I clinical trial using recombinant vaccinia viruses encoding human B7.1 (rV-B7.1), three of 12 patients demonstrated disease control with one patient achieving a durable response lasting more than 59 months, which was associated with the emergence of melanoma-specific memory T cell responses and autoimmune vitiligo [3]. In a second phase I clinical trial using recombinant vaccinia viruses encoding a triad of T cell co-stimulatory molecules including B7.1, ICAM-1, and LFA-3 (rV-TRICOM), a 30.7% objective response rate was demonstrated with one patient achieving a durable complete response [4]. Ideally, a predictive biomarker to identify patients more likely to respond to treatment or to identify patients likely to benefit from continued therapy is vital.

In order to identify putative biomarkers of response to oncolytic vaccinia virus immunotherapy, we developed a customized microarray focused on gene expression changes in T cells following exposure to rV-infected autologous dendritic cells. This strategy was undertaken because of the previously established relationship between therapeutic responses and T cell responses observed in early phase clinical trials [3,5]. We identified 1,261 genes significantly modulated in T cells exposed to vaccinia virus, and from those we chose to focus on one biomarker, immunoglobulin-like transcript 2 (ILT2) because of its known function in suppressing T cell responses. Furthermore, this biomarker is easy to monitor due to its expression on the surface of T cells. The data reported here support the potential role of ILT2 as a biomarker of response to oncolytic vaccinia virus immunotherapy in patients with melanoma. Larger prospective clinical trials will be needed to further validate ILT2 as a general predictive biomarker for responses using oncolytic virus treatment and possibly other forms of tumor immunotherapy.

## Results

### Biomarker discovery for oncolytic vaccinia virus immunotherapy responses

**Figure 1:**
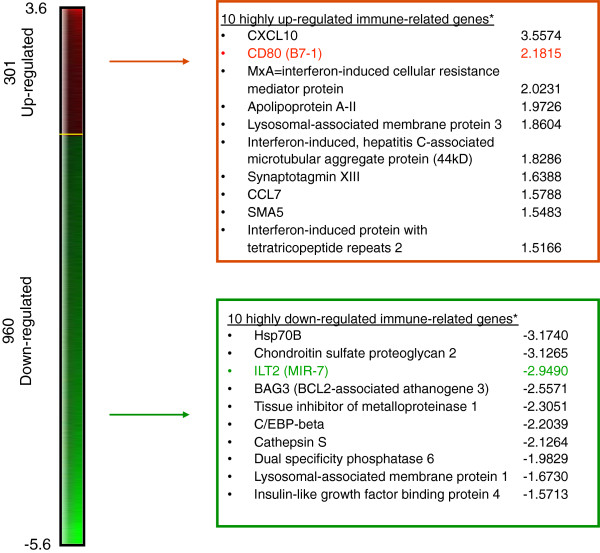
**Genes differentially expressed in healthy donor PBMCs after rV-B7.1 infection.** Healthy donor (n = 6) PBMCs were treated with rV-B7.1 vaccine for 12 hours and gene expression profiles were analyzed using a customized 32x24x23 (~17,000 spot) human cDNA microarray. Ten immune-related genes of interest showing high degrees of up-regulation and ten immune-related genes showing high degrees of down-regulation are listed along with their intensity ratios. All genes tested are listed in Additional files 1 and 2. *P < 0.05.

To identify putative biomarkers predictive of response with oncolytic vaccinia virus immunotherapy, healthy donor (n = 6) PBMCs were treated with rV-B7.1 vaccine for 12 hours and gene expression profiles were analyzed by gene microarray. The analysis identified 301 up-regulated and 960 down-regulated genes following treatment (Additional file 1: Table S1 and Additional file 2: Table S2). Ten highly up-regulated and ten highly down-regulated immune-related genes of interest are listed in Figure 1. The majority of the highly up-regulated genes were interferon-gamma-related factors, including B7.1 (CD80) consistent with the potent interferon-gamma response elicited by vaccinia viruses [6]. We chose to focus on ILT2 because: 1) ILT2 gene expression was significantly down-regulated by rV-B7.1 among the 960 identified down-regulated genes; 2) ILT2-related factors, such as ILT3, have been associated with inhibiting immune-mediated tumor rejection [7]; 3) ILT gene expression is inversely regulated by B7.1; and 4) ILT2 is known to be an inhibitor of T cell activation [8]. Thus, we hypothesized that ILT2 could be a potential biomarker and/or target for immunotherapy.

**Figure 2:**
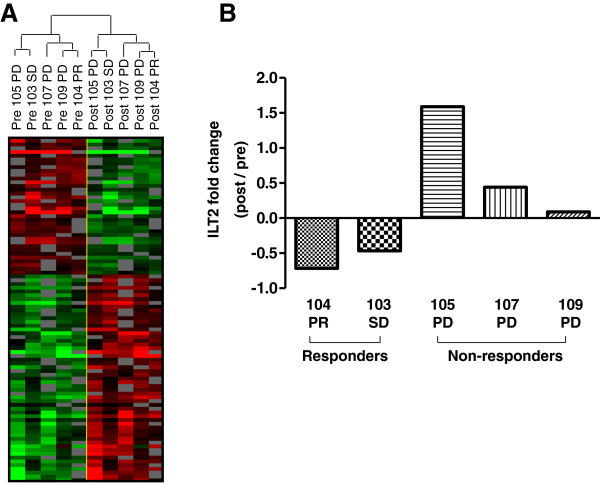
**Genes differentially expressed in patients’ tumor microenvironment before and after rV-B7.1 treatment.** Gene expression profiles from metastatic melanoma lesions were obtained from fine needle aspiration (FNA) samples (n = 5) before (pre) the first and four weeks after (post) the third intra-tumoral rV-B7.1 vaccination and analyzed using a customized 32x24x23 (~17,000 spot) human cDNA microarray. **(A)** The resultant heat map is shown. **(B)** Fold change (post / pre) of ILT2 gene expression in tumor FNAs from **(A)** shown for responders (n = 2) and non-responders (n = 3). PD = progressive disease, SD = stable disease, PR = partial response.

Gene expression profiles from metastatic melanoma lesions were obtained from fine needle aspiration samples before the first and four weeks after the third intra-tumoral rV-B7.1 vaccination from five patients (two responders and three non-responders) and analyzed by customized gene microarray. The resultant heat map is shown in Figure 2A. We identified 55 genes that were up-regulated and 37 genes that were down-regulated following oncolytic virus injection (Additional file 3: Table S3). Among these genes, ILT2 was down-regulated in the tumor of clinically responding patients and up-regulated in non-responders (Figure 2B), suggesting that ILT2 may be a predictive biomarker of response to oncolytic vaccinia virus treatment.

### Detection of ILT2 expression on T cells of melanoma patients

**Figure 3:**
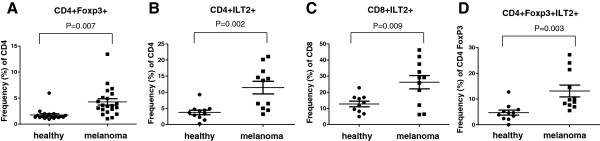
**ILT2 expression by T cell populations of healthy donors and metastatic melanoma patients. (A)** Evaluation by flow cytometry of frequency of peripheral blood cells expressing FoxP3 among CD4+ T cells (healthy donors n = 19, melanoma patients n = 21) and (B-D) ILT2 among **(B)** CD4+ T cells (healthy donors n = 11, melanoma patients n = 11), **(C)** CD8+ T cells (healthy donors n = 10, melanoma patients n = 11), and **(D)** CD4+FoxP3+ T cells (healthy donors n = 11, melanoma donors n = 11).

In order to determine the baseline level of ILT2 expression in patients with melanoma, we evaluated ILT2 expression by flow cytometry in healthy donors and patients with metastatic melanoma. Since ILT2 is commonly expressed on T cells, we focused our analysis on ILT2 expression on total T cell populations and T cell regulatory/suppressor subsets. First, we evaluated the frequency of CD4+FoxP3+ Tregs in melanoma patients and healthy donors, and as previously reported [9], we found an increase in Tregs in the melanoma patients compared to healthy donors (n = 21 and 19, respectively); P = 0.007; Figure 3A). We next evaluated the mean percentage of CD4+ and CD8+ T cells expressing ILT2 and found it to be significantly elevated in metastatic melanoma patients compared to healthy donors (n = 11 and 10-11, respectively; P = 0.002 and P = 0.0009, respectively; Figure 3B and C). We also found that the frequency of CD4+FoxP3+ Tregs expressing ILT2 was higher in patients with melanoma compared to healthy donors (n = 11 and 11, respectively; P = 0.003; Figure 3D). These data demonstrate that baseline levels of ILT2 can be identified in peripheral T cells and that patients with metastatic melanoma have significantly higher frequencies of T cell subsets expressing ILT2.

### Detection of ILT2 expression on T cells from oncolytic vaccinia virus-treated patients

**Figure 4:**
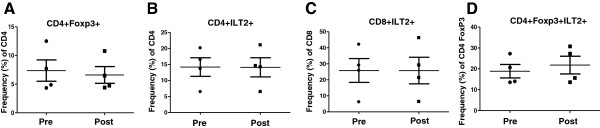
**ILT2 expression by T cell populations in melanoma patients before and after rV-B7.1 treatment.** (A**-**D) Frequency of peripheral blood cells expressing **(A)** FoxP3 among CD4+ T cells and ILT2 among **(B)** CD4+ T cells, **(C)** CD8+ T cells, and **(D)** CD4+FoxP3+ T cells was evaluated by flow cytometry in metastatic melanoma patients (n = 4) before (pre) and after (post) intra-tumoral rV-B7.1 treatment.

**Figure 5:**
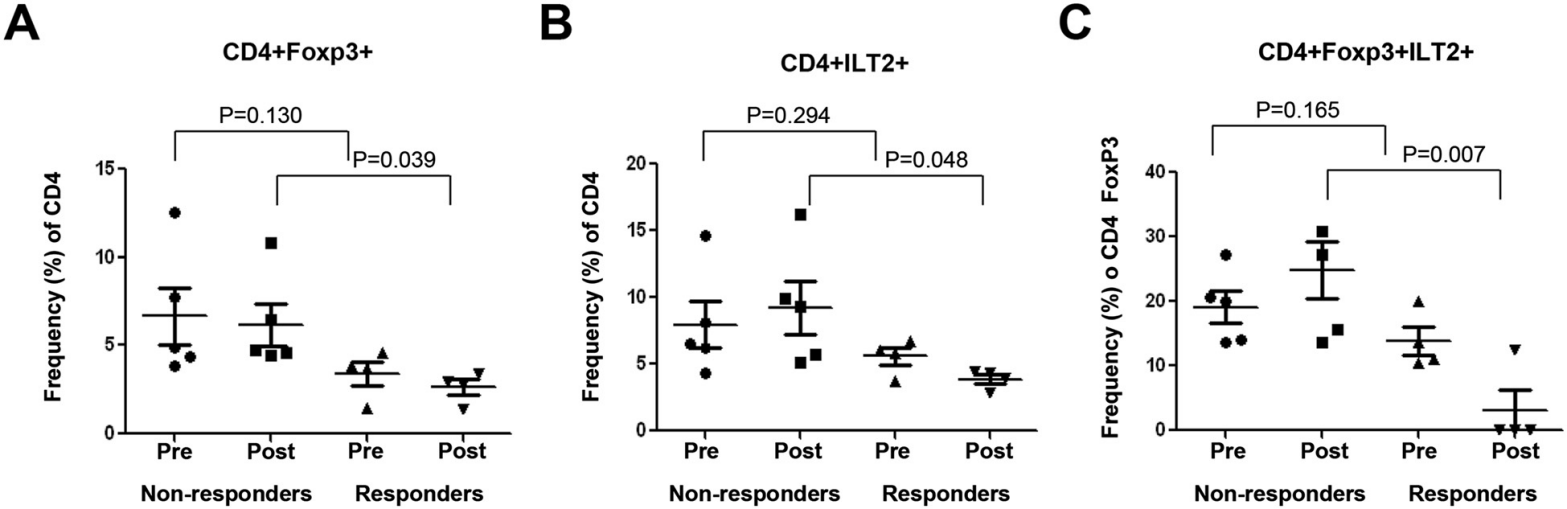
**ILT2 expression by CD4+ T cell populations in non-responder and responder melanoma patients before and after rV-B7.1 treatment.** (A**-**D) Frequency of peripheral blood cells expressing **(A)** FoxP3 among CD4+ T cells and ILT2 among **(B)** CD4+ T cells, and **(C)** CD4+FoxP3+ T cells was evaluated by flow cytometry in non-responder (n = 5) and responder (n = 4) metastatic melanoma patients before (pre) and after (post) intra-tumoral rV-B7.1 treatment.

First, we analyzed if there were any differences in ILT2 expression on CD4+, CD8+, Treg or Ts cells among all patients (responders and non-responders) before and after intra-tumoral treatment with oncolytic vaccinia and found no significant differences (Figure 4). Next, we compared ILT2 expression in the various T cell subsets in PBMCs from clinical responder and non-responder patients. Figure 5 displays the data for CD4+ T cells in which we observed no difference in baseline (pre-treatment) Tregs (P = 0.130) but did see a significantly decreased frequency of post-treatment Tregs in responding versus non-responding patients (P = 0.039, Figure 5A). We saw a similar pattern in CD4+ILT2+ T cells with no difference prior to treatment across all patients (P = 0.294), but a significantly lower frequency of CD4+ILT2+ T cells was detected following treatment in responding versus non-responding patients (P = 0.048; Figure 5B). Since ILT2 could potentially be a previously unrecognized marker of Treg cells we also stained CD4+ T cells for both FoxP3 and ILT2. While there were no differences in baseline CD4+FoxP3 + ILT2+ T cells in all patients (P = 0.165), the responding patients demonstrated a significantly lower frequency in this population following treatment with oncolytic vaccinia virus (P = 0.007; Figure 5C).

**Figure 6:**
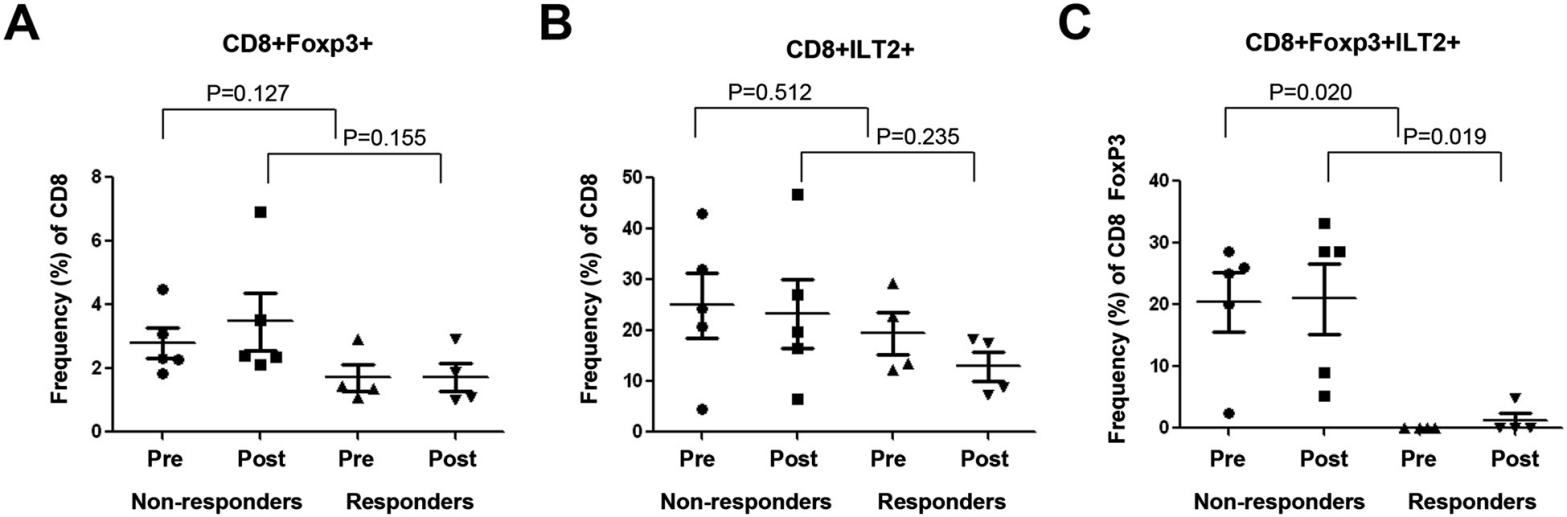
**ILT2 expression by CD8+ T cell populations in non-responder and responder melanoma patients before and after rV-B7.1 treatment.** (A**-**D) Frequency of peripheral blood cells expressing **(A)** FoxP3 among CD8+ T cells and ILT2 among **(B)** CD8+ T cells, and **(C)** CD8+FoxP3+ T cells was evaluated by flow cytometry in non-responder (n = 5) and responder (n = 4) metastatic melanoma patients before (pre) and after (post) intra-tumoral rV-B7.1 treatment.

We also evaluated CD8+ Ts cells and found that there were no differences in these cells at baseline between responding and non-responding patients (P = 0.127, Figure 6A). Evaluation of CD8+ILT2+T cells showed a similar pattern with no overall differences between responding and non-responding patients pre (P = 0.512) or post (P = 0.235) treatment (Figure 6B). However, when we analyzed CD8+FoxP3+ILT2+ T cells we found a significantly lower frequency of these cells in responding patients at baseline (P = 0.020) and after treatment (P = 0.019; Figure 6C) than in non-responding patients. These data suggest that ILT2 may be useful for identifying a subset of CD8+ suppressor T cells that may be a predictive biomarker of oncolytic virus immunotherapy outcome.

## Discussion

Oncolytic virus therapy is gaining momentum as a novel form of immunotherapy for patients with cancer. Vaccinia virus has been a highly versatile viral vector and was initially developed for vaccination of cancer patients by encoding tumor-associated antigens alone or with T cell co-stimulatory molecules. In contrast to these agents, vaccinia viruses encoding only T cell co-stimulatory molecules were generated for use as oncolytic agents wherein the viruses are injected directly into tumors resulting in a direct oncolytic effect, release of individual tumor-specific antigens and local expression of T cell co-stimulatory molecules to enhance the induction and activation of local T cell responses [3,4]. Specifically, in a series of early phase studies we demonstrated the safety and initial therapeutic responses of vaccinia viruses encoding human T cell co-stimulatory molecules (rV-B7.1 and rV-TRICOM). Other approaches utilizing modified herpes viruses, native coxsackie viruses and Newcastle disease viruses have also reported promising results [1]. The identification of predictive biomarkers is important for optimizing patient selection for treatment, monitoring individual immune responses in patients undergoing active treatment, and determining which patients may achieve clinical responses with continued therapy. Immunotherapy has been associated with delayed kinetics of therapeutic responses, as reported for the anti-CTLA-4 monoclonal antibody therapy with ipilimumab [10], making the discovery of predictive biomarkers critical for the clinical management of patients receiving immunotherapy. This is the first report of a potential biomarker for oncolytic virus immunotherapy, although the results may have implications for other forms of immunotherapy as well.

We identified ILT2 as a putative biomarker from a customized microarray performed on peripheral blood mononuclear cells exposed to rV-B7 oncolytic virus and confirmed the marker in a second microarray (Figure 2A) and quantitative PCR (Figure 2B) analysis from fine needle aspirate samples taken from tumors injected with the oncolytic rV-B7 and rV-TRICOM viruses. ILT2 is an immune regulatory receptor that contains an extracellular Ig-like domain, is responsible for ligand binding at the cell surface, and has a long cytoplasmic tail containing immunoreceptor tyrosine inhibitory motif (ITIM), which recruits inhibitory phosphatases and mediates inhibition of cell activation intracellularly [11-14]. ILT2 is expressed on T cells and antigen-presenting cells where it inhibits T cell receptor signaling and T cell activation and proliferation. ILT2 interacts with HLA-G, expressed on antigen-presenting cells and tumor cells, such as melanoma [15-18]. ILT2 functions to dampen T cell responses and may contribute to T cell tolerance in cancer, although the role of ILT2 in oncology has not been well studied. T cell co-inhibitory molecules, such as CTLA-4 and PD-1, have been validated through numerous pre-clinical and clinical trials, as critical mediators of tumor immune suppression [19]. We speculate that ILT2 may represent another T cell co-inhibitory receptor with similar activity in patients with cancer.

In this study, we found that ILT2 could be detected on both CD4+ and CD8+ T cells in patients with metastatic melanoma and that ILT2 correlated with FoxP3 expression. While this further supports the potential role of ILT2 in inhibiting anti-tumor immunity, this association needs to be further validated in larger, prospective trials. Whereas FoxP3 is an intracellular protein, ILT2 is an extracellular membrane protein that is easily detected by flow cytometry without disruption of the cell membrane. Importantly, we observed a significant increase in ILT2-expressing T cells in melanoma patients compared to healthy donors (Figure 3) and this is similar to previous reports of increased frequency of Tregs in patients with melanoma [9].

We did not see significant differences in ILT2-expressing T cell or Treg populations following oncolytic vaccinia immunotherapy (Figure 4) but we did find that ILT2-expressing T cells were correlated with clinical response as defined through local gene expression in the tumor microenvironment (Figure 2B) and in peripheral blood T cell staining (Figures 5 and 6). These data suggest that oncolytic virus therapy may not be changing the T cell responses but rather ILT2 expression may be a more general biomarker of patients predisposed to respond to immunotherapy. This effect was seen in both CD4+ and CD8+ T cells, and a low frequency of CD8+ILT2+ T cells appeared to be highly predictive of clinical response even at baseline (Figure 6C).

## Conclusions

We have identified ILT2 as a potential biomarker of clinical response in melanoma patients treated with oncolytic vaccinia virus immunotherapy. Patients with a decrease in CD4+ILT2+ T cells, CD4+FoxP3+ILT2+ T cells, and CD8+FoxP3+ILT2+ T cells following treatment had an increased likelihood of responding to oncolytic immunotherapy. While this study identified ILT2 as a putative biomarker, further validation of ILT2 will require evaluation in larger, prospective clinical trials. Our data also suggests that decreases in ILT2-expressing T cells were predictive of clinical response but not of exposure to oncolytic viruses and this may have implications for ILT2 as a more general predictive biomarker of response to tumor immunotherapy. Finally, the general role of ILT2 as a T cell inhibitor has not been widely explored but it is possible that ILT2 may be a novel T cell checkpoint target for immunotherapy based on its function as a T cell co-inhibitory receptor.

## Methods

### Patient characteristics

The patient characteristics and clinical trial results associated with the patient samples used here have been previously reported [3,4]. These clinical trials were approved by the Institutional Review Board of Columbia University, NY. Briefly, patients with metastatic melanoma and accessible, measurable lesions received three intra-tumoral (IT) injections every four weeks. In one phase I clinical trial, 12 patients with metastatic melanoma and accessible lesions for injection were treated in a dose escalation manner with two different concentrations of rV-B7.1 (4.26 × 10^7^ and 4.26 × 10^8^ plaque-forming units [PFUs]). In a second phase I clinical trial, 13 patients with accessible, metastatic melanoma were treated in a dose escalation manner with rV-TRICOM (5.1 × 10^6^ and 5.1 × 10^8^ PFUs). Prior to each injection and two weeks after injection, peripheral blood was collected for serum and T cell assays. Four weeks after the third injection, patients underwent imaging studies to assess disease status and fine needle aspiration of injected lesions was performed on those patients who voluntarily consented. Those patients without objective disease progression and who still met eligibility criteria were offered booster vaccinations following the same schema. Written informed consent was obtained from all patients and the trial was conducted in accordance with the Declaration of Helsinki. Six healthy donors were solicited from local institutions and had peripheral blood collected after providing written informed consent.

### Gene expression profiling

To compare gene expression profiles between responders (n = 2) and non-responders (n = 3), tumor fine needle aspirates (FNAs) were obtained. RNA was extracted from the FNAs for microarray testing. PBMCs from six healthy donors were treated with rV-B7.1 and used as controls. RNA was harvested from PBMCs before and 12 hours after treatment. The 32x24x23 (17,000 spot) human cDNA microarray was prepared in the Immunogenetics Section of the Department of Transfusion Medicine, Clinical Center, National Institutes of Health [20,21]. Clones used for the printing of 17 k cDNA array included a combination from a RG_HsKG_031901 7 k clone set and 10,000 clones from the RG_Hs_seq_ver_070700 40 k clone set (Research Genetics, Huntsville, AL), which were designed to cover genes relevant to immune function. The cDNA clones include 12,072 uniquely named genes, 875 duplicates of named gene and the remainder consists of expression sequence tags. Reproducibility of the data set was assessed by a matrix of repeated experiments. A level of concordance of gene expression ≥ 95% was met in all experiments. Analysis of array data was based on Cluster and Treeview programs from the Stanford Genome Analysis Group Software package (Stanford, CA).

### Flow cytometry analysis

To determine the frequency of T cells and regulatory/suppressor T cells expressing ILT2, FITC-CD4, PE-CD8, PECY5-CD3, APC-ILT2 and eFluor450-Foxp3 antibodies (Ebioscience, San Diego, CA) were used. Data acquisition and analysis were performed using a FACSCantoII flow cytometer (BD Bioscience, San Jose, CA) and FlowJo software (TreeStar, Ashland, OR).

### Statistical analysis

Results are expressed as mean ± standard deviation (SD) or range when appropriate. A student t test was used in comparisons of two groups. One-way analysis of variance (ANOVA) was applied (with Bonferonni correction) when comparing three or more groups. Differential gene expression between pre- and post-rV-B7.1 treatment samples was analyzed by two-tailed, paired t test. Statistical analyses were performed using Prism 5 software (Gaphpad Software, Inc., La Jolla, CA). Values of P < 0.05 were considered statistically significant.

## Abbreviations

ILT2: Immunoglobulin-like transcript 2; PBMC: Peripheral blood mononuclear cell; rV-B7.1: Recombinant vaccinia viruses encoding human B7.1; rV-TRICOM: Recombinant vaccinia viruses encoding a triad of T cell co-stimulatory molecules including B7.1, ICAM-1, and LFA-3; rV: Recombinant vaccinia viruses; Treg: CD4+FoxP3+ regulatory T cell; Ts: CD8+FoxP3+ suppressor T cell.

## Competing interests

The authors have no financial conflicts of interest to report.

## Authors’ contributions

HLK conceived and designed the clinical trial; HLK treated and evaluated patients; VM, and FMM provided study materials; DWK, SKS, and VM processed samples and analyzed immune responses; AZ, VM, DWK, SKS, FMM, MCJ, and HLK analyzed and interpreted data; AZ, DWK, SKS, and HLK performed statistical analysis of the data; AZ, MCJ, DWK, SKS, and HLK and wrote the manuscript. All authors have agreed to the content of this manuscript, including the data as presented. All authors read and approved the final manuscript.

## Supplementary Material

Additional file 1: Table S1 Up-regulated genes in PBMCs after rV-B7.1 treatment. Healthy donor (n = 6) PBMCs were treated with rV-B7.1 vaccine for 12 hours and gene expression profiles were analyzed using a customized 32x24x23 (~17,000 spot) human cDNA microarray. Click here for file

Additional file 2: Table S2 Down-regulated genes in PBMCs after rV-B7.1 treatment. Healthy donor (n = 6) PBMCs were treated with rV-B7.1 vaccine for 12 hours and gene expression profiles were analyzed using a customized 32x24x23 (~17,000 spot) human cDNA microarray. Click here for file

Additional file 3: Table S3 Up-regulated and down-regulated genes in the tumor microenvironment after rV-B7.1 treatment. Gene expression profiles from metastatic melanoma lesions were obtained from fine needle aspiration (FNA) samples (n = 5) before (pre) and after (post) rV-B7.1 vaccination and analyzed using a customized 32x24x23 (~17,000 spot) human cDNA microarray. Results pre and post oncolytic vaccine treatment are further shown for responders (n = 2; 103 SD and 104 PR) and non-responders (n = 3; 105 PD, 107 PD, 109 PD). Click here for file
